# An Immunohistochemical Anomaly: A Case Report and Systematic Review of Myofibroblastoma of the Breast

**DOI:** 10.7759/cureus.46125

**Published:** 2023-09-28

**Authors:** Mohamad Kaki, Sarah Klein, Chinar Singh, Breanne Kothe, Jose Martin

**Affiliations:** 1 Dr. Kiran C. Patel College of Osteopathic Medicine, Nova Southeastern University, Davie, USA; 2 Dr. Kiran C. Patel College of Osteopathic Medicine, Nova Southeastern University, Clearwater, USA; 3 Dr. Kiran C. Patel College of Osteopathic Medicine, Nova Southeastern University, Fort Lauderdale, USA; 4 General Surgery, Broward Health, Tamarac, USA

**Keywords:** immunohistochemical, diagnostic mammography, lumpectomy, benign breast tumor, myofibroblastoma

## Abstract

Myofibroblastoma (MFB) is a rare but benign mesenchymal tumor most commonly appearing within breast tissue. Most cases of MFB occur in postmenopausal women and are treated by surgical excision. The diagnosis of MFB is made through immunohistochemical (IHC) analysis, with the most common biomarkers being CD34+, desmin+, smooth muscle actin+, and vimentin+. In this article, we describe a case of an MFB in a premenopausal female with variance from classic IHC findings. We also performed a systemic review of the MFB of the breast. The systemic review compiles the most common IHC findings of MFB, patient demographics, treatment methods, lesion size, and the presence or absence of pain associated with the lesion. As MFB can share many features with other breast lesions, including potentially malignant ones, this article sought to underline the most common IHC findings and characteristics of MFB to aid in the proper diagnosis of MFB.

## Introduction

Myofibroblastoma (MFB), though benign, is a rare mesenchymal tumor most commonly found in the breast [1-3]. Histopathology of classic MFB demonstrates lesions composed of spindle cells and is difficult to differentiate from malignant breast pathology [1,4]. Though most commonly found in the breast, MFB can also occur in various extramammary locations, including the head, neck, popliteal fossa, groin, vulva, and paratesticular region [1]. Many lesions are found incidentally on screening mammography or computerized tomography (CT) scan and typically present as a firm, mobile, and well-demarcated ovoid lesion with surrounding pseudocapsule [1]. Upon identification of the suspicious lesion, core-needle tissue biopsy with thorough histopathologic and immunohistochemical (IHC) analysis is required for accurate diagnosis of the tumor [1,3-5]. Though histopathologic analysis is very detailed, diagnosis of MFB can still be very difficult as it is histologically similar to many breast malignancies [1]. Additionally, there are many known variations of the classic tumor, including cellular, collagenous, decidua-like, epithelioid, infiltrative, lipomatous, and myxoid variants, further complicating diagnosis [1,4,6,7].

MFB is far more common in men than in women, and subsequent surgical management varies based on tumor location and gender [1,2]. After meticulous histopathologic characterization of tissue biopsies from the tumor confirms the preliminary diagnosis of MFB, surgical management is typically initiated [1,6]. Total excision is curative and therefore the management of choice, and excision is planned based on the location of the lesion [3,5,8]. For extramammary lesions, care is taken to properly excise the tumor from the surrounding anatomy and does not vary significantly across genders; however, surgical management of breast pathology differs due to avoidance of disfiguring deformity of the female breast [1]. Total mastectomy is typically planned for males with findings consistent with MFB, especially in the presence of gynecomastia [1]. For females, lumpectomy is usually the surgical treatment of choice as it often results in the preservation of the majority of breast tissue and has a less detrimental impact on the psychosocial health of the patient [1].

## Case presentation

History

A 47-year-old premenopausal female presented to the clinic for evaluation of a breast mass. The patient first noticed this mass one year before our initial evaluation. On presentation to our clinic, the patient admitted associated pain with the breast mass but denied any breast discharge. She denied any family history of breast cancer.

The patient had a past medical history of chronic leukopenia, chronic abdominal pain, prediabetes, left eye cataract, and resolved dyslipidemia (according to previous records obtained). Her past surgical history was significant for cataract surgery and tubal ligation.

Diagnosis

On physical examination, a small, palpable mass was found at the 9 o’clock position on the right breast. No discharge or nipple retractions were appreciated on examination. No other masses were appreciated. No axillary lymphadenopathy bilaterally was found.

**Figure 1:**
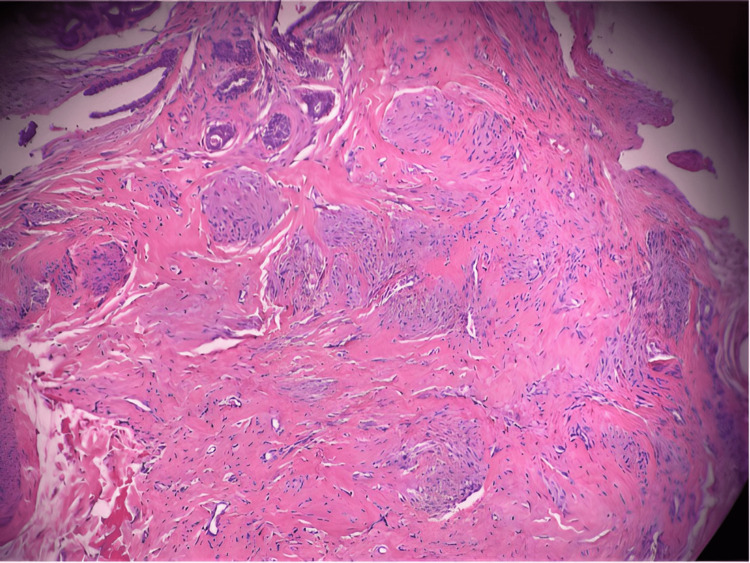
Histologic representation of proliferation of myofibroblasts. Bland darkly stained clusters of myofibroblast nuclei can be seen in this slide.

**Figure 2:**
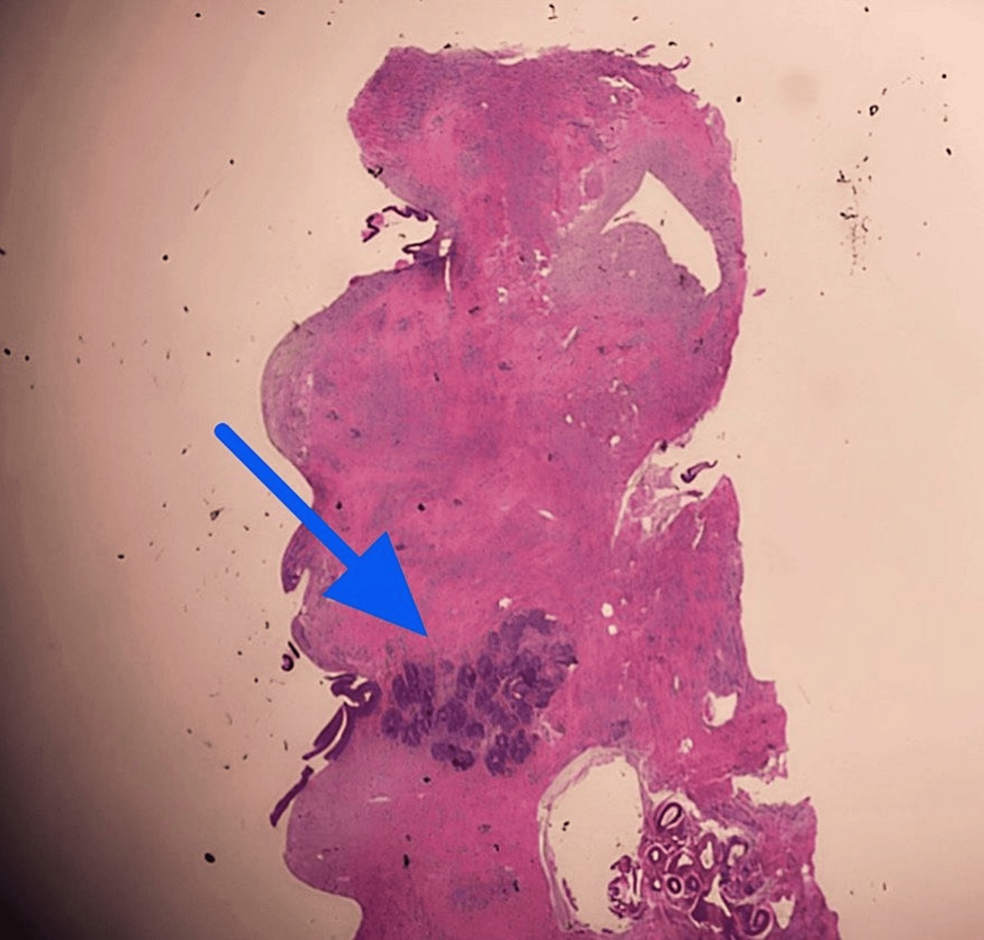
The proliferation of myofibroblasts. The patient’s biopsy has cellular stroma in between ducts (blue cells in the center), which are spindle-like cells that are proliferating.

**Figure 3:**
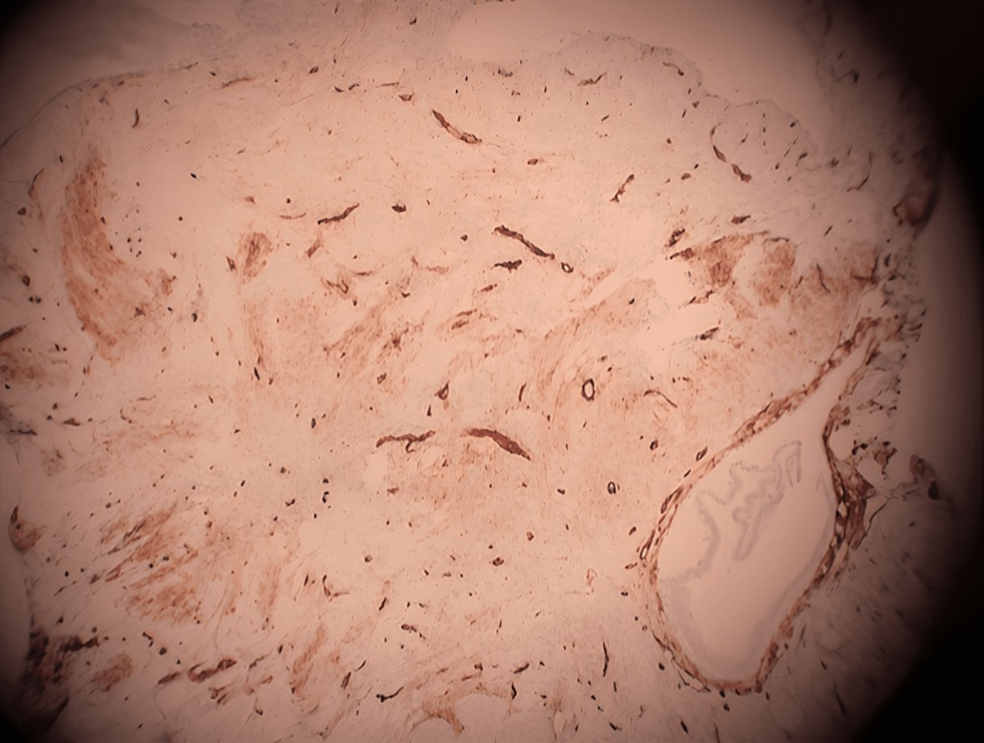
Immunohistochemical stain for CD34-positive cells.

**Figure 4:**
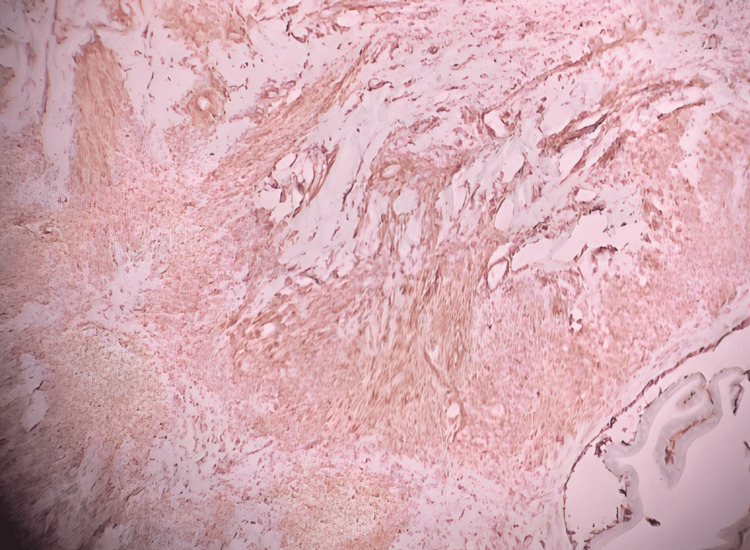
Representation of Immunohistochemical staining for smooth muscle actin-positive cells.

Access to the patient’s medical records showed that the patient had imaging completed before her first office visit with us. Three months before her office visit with us, the patient had a right breast mammogram which showed two new 1 cm mass lesions, one within the right breast at the 9 o’clock axis 2 cm from the nipple, and the other at the 9 o’clock axis 6-8 cm from the nipple. Two months before her office visit with us, the patient underwent a right breast ultrasound, where findings included a 1 cm hypoechoic solid mass lesion at the 9 o’clock axis, and a 9 mm cluster of cysts present 5 cm from the nipple at the 9 o’clock axis, findings which were consistent with her previous mammogram. A core needle biopsy was recommended due to these findings. Approximately one month later, the patient underwent ultrasound-guided, vacuum-assisted right breast core needle biopsy of the 1 cm lesion 2 cm from the nipple at the 9 o’clock position with tissue marker placement. Gross evaluation of the patient’s lesion obtained via biopsy revealed formalin-fixed multiple fragments of yellow-tan fibrofatty tissue 1.5 × 1.5 × 0.4 cm in aggregate. Pathophysiology of the lesion showed IHC for pan-cytokeratin, desmin, and estrogen receptor (ER) was negative, while CD34, vimentin, and smooth muscle actin were positive (Figures 1-4). These results indicated a spindle cell lesion suggestive of MFB. Complete surgical excision was recommended due to these results, and the patient was referred to our office for this reason.

Treatment

Upon presentation to our clinic, after a complete history and physical examination, conservative but complete resection of the mass was recommended to the patient. She was scheduled for a SAVI Scout-guided right breast lumpectomy. The risks, benefits, and alternatives of the procedure were explained to the patient. The patient verbalized understanding and provided informed consent. She underwent a successful lumpectomy with confirmation of MFB. The patient was subsequently lost to follow-up.

## Discussion

MFB of the breast is a rare benign tumor of mesenchymal tissue. Most reported cases of MFB of the breast involve men and postmenopausal women [9]. Some cases, including ours, have involved MFB appearing in premenopausal women [10]. While the patient presented in this case study had complained of pain associated with her breast lesion, most cases typically present as painless and are overall asymptomatic [11]. A physical examination of MFB usually reveals a solid, well-defined, mobile lesion without adherence to the dermis. Clinically, MFB shares many features with those of benign breast fibroadenoma which makes the differentiation between the two challenging and underlines the importance of core needle biopsy and histopathological evaluation to aid in the diagnosis of MFB.

The diagnosis of MFB begins with mammography and ultrasonography, which typically reveal a well-defined, slightly hypoechoic solid, oval-like mass of varying sizes [12]. Because imaging cannot differentiate between MFB and other benign breast lesions, these findings should be followed up with core needle biopsy and histopathological examination, which remains the gold standard for diagnosis of MFB [11]. IHC of the lesion was positive for CD34, vimentin, and smooth muscle actin. The lesion was negative for cytokeratin, desmin, and ER. The absence of certain epithelial tissue such as cytokeratin helps differentiate MFB from potentially malignant lesions [13]. IHC seen in most cases of MFB reported in the literature are ER-positive; however, some cases have shown MFB to be ER-negative, including ours [13].

Although benign, MFB lesions are typically surgically excised due to their ability to continue growing and potentially compress nearby tissue and structures. As all reported cases in the literature have involved the removal of the lesion, the long-term implications cannot be fully understood [14]. While surgical excision is seen in nearly all of the cases of MFB in the literature, Fakim et al. described the use of a vacuum-assisted excision technique and advocated for this technique in smaller lesions as it proved to be a safer and cheaper option than surgical excision [7]. Modified radical mastectomy has also been reported in the literature as a treatment for MFB. One case misinterpreted a breast lump as invasive lobular carcinoma due to histological findings and IHC showing strong positivity of estrogen and progesterone receptors (ER/PR+) [15]. The patient subsequently underwent a modified radical mastectomy for treatment of the presumed invasive lobular carcinoma; however, upon gross examination and large IHC profiling, the diagnosis of MFB was obtained [15]. Out of the 38 cases reviewed in our study, only one case documented a diagnosis of MFB with a negative ER seen on the IHC panel. Sixteen positives were documented while the rest of the cases included neither the presence nor absence of ER. While the presence of CD34 is often described in the literature as the defining IHC marker, we have noted seven cases that reported MFB with negative CD34 markers. All cases but one that documented staining for protein S-100 were negative. The lack of S-100 helps differentiate MFB from other lesions such as neurofibroma or other tumors of neural origin which show S-100 positive reactivity [16]. The median age of diagnosis of MFB was 59 years old. It is worth mentioning that the youngest age of diagnosis of MFB was 17 years old, which illustrates the possibility of MFB appearing in this age group. This finding emphasizes the importance of IHC in the proper diagnosis of MFB to achieve proper management.

Methods and search strategy

We conducted an electronic-based search using the database PubMed. The medical subject heading “myofibroblastoma of the breast” was used. The search was limited to cases in humans and published in the English language with full text available, with no restriction based on the year published. We manually searched the reference lists of identified studies. We included all original case report articles and excluded cases that were not relevant to MFB of the breast.

Results

**Figure 5:**
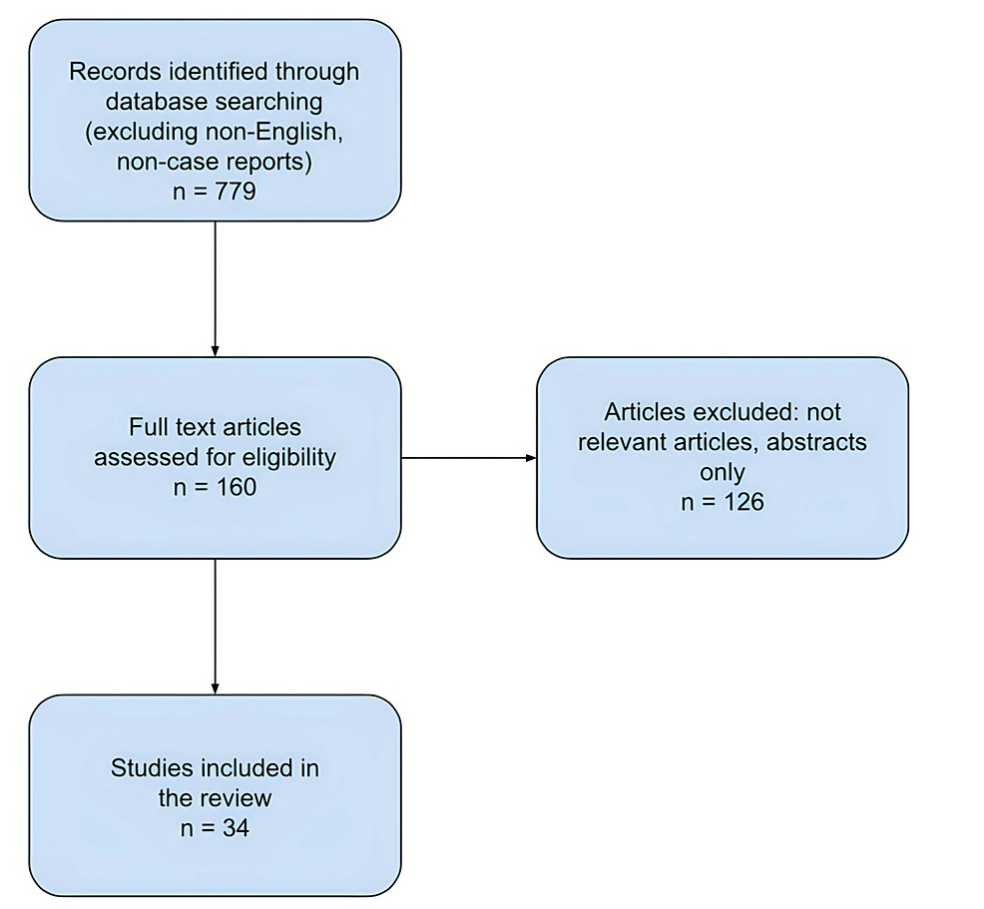
Flow diagram for article selection.

Our preliminary search for “myofibroblastoma AND breast” yielded 779 citations with exclusion criteria of non-English articles and non-case report articles. After excluding articles that did not have the full text freely available or available through Nova Southeastern University’s library, 160 articles remained. The remaining articles were then manually reviewed and after eliminating articles that were not pertinent to MFB of the breast tissue, were not case reports, or were not accessible by the authors, 34 articles remained. See Figure 5 for a flowchart illustrating the article selection process. Additionally, four articles reported on multiple cases, yielding a total of 38 patient case reports analyzed. Raw data are available in Table 1. Summarized comparison data across 38 reported cases of MFB of the breast in patient presentation and immunohistochemistry are presented in Table 2 and Table 3, respectively.

**Table 1:** Characteristics of all studies included in the systematic review.

Reference number	Author	Age	Sex	Associated Pain	CD34	Vimentin	Smooth Muscle Actin	ER	Pancytokeratin	Desmin	S100	Size of lesion	Treatment
[1]	Shanmugasiva et al., 2018	80	M	No	Positive	-	Negative	Positive	Negative	Negative	Negative	3.6 cm	Lumpectomy
[3]	Aytac et al., 2015	64	F	No	Positive	Positive	Negative	Positive	-	Positive	-	3 cm	Lumpectomy
[4]	Mečiarová et al., 2023	48	F	No	Negative	Positive	Positive	Positive	Negative	Positive	Negative	4 cm	Lumpectomy
[7]	Fakim et al., 2019	52	F	No	Negative	-	Positive	Positive	Negative	Positive	Negative	0.8 cm	Vacuum-assisted excision
[11]	Akrami et al., 2019	65	M	No	Positive	-	-	Positive	Negative	Positive	-	4 cm	Modified radical mastectomy
[14]	Saffar et al., 2021 (case 1)	52	F	No	Positive	-	Positive	-	-	-	-	2.5 cm	Lumpectomy
[14]	Saffar et al., 2021 (case 2)	75	M	-	Positive	-	Positive	-	-	Positive	-	-	Lumpectomy
[14]	Saffar et al., 2021 (case 3)	55	F	No	Positive	-	-	Positive	Negative	-	Negative	3 cm	Lumpectomy
[15]	Talwar et al., 2021	65	F	No	Positive	-	-	Positive	Negative	Positive	-	3.5 cm	Modified radical mastectomy
[17]	Allahverdi et al., 2017	61	M	No	Positive	-	Negative	-	Negative	-	Negative	8 cm	Lumpectomy
[18]	El Amine et al., 2016	17	F	No	Positive	-	-	-	-	-	Negative	2 cm	Lumpectomy
[19]	Angelico et al., 2021	80	M	No	Positive	Positive	Negative	Positive	Negative	Positive	Negative	2.5 cm	Lumpectomy
[20]	Bakuła-Zalewska et al., 2012	65	F	No	Positive	-	Positive	-	Negative	Positive	-	6 cm	-
[21]	Barbuscia et al., 2013	82	F	-	Positive	Positive	-	Positive	Negative	Negative	-	10 cm	Unilateral mastectomy
[22]	Baxendine-Jones et al., 2001 (case 1)	60	F	-	Positive	Positve	Positive	-	Negative	Positive	Negative	2 cm	-
[22]	Baxendine-Jones et al., 2001 (case 2)	51	F	-	Negative	Positive	Positive	-	Negative	Positive	Negative	1 cm	-
[23]	Bharathi et al., 2014	45	F	No	Positive	Positive	-	-	Negative	-	-	8 cm	Lumpectomy
[24]	Boudaouara et al., 2017	43	F	No	Negative	Positive	Positive	-	Negative	Positive	Positive	2 cm	Lumpectomy
[25]	Desrosiers et al., 2007 (case 1)	63	M	-	Positive	-	Positive	Positive	-	Positive	Negative	0.9 cm	-
[25]	Desrosiers et al., 2007 (case 2)	66	M	-	Positive	-	Positive	Positive	-	Positive	Negative	1.9 cm	-
[26]	Fatani et al., 2023	76	F	No	Negative	-	-	-	Negative	Positive	Negative	5.5 cm	Lumpectomy
[27]	Gaetano et al., 2018	58	F	No	Positive	Positive	Negative	Negative	Negative	-	-	2 cm	Lumpectomy
[28]	Gurzu et al., 2012	75	M	No	Positive	Positive	Positive	Positive	Negative	Positive	Negative	1.0 cm	Lumpectomy
[29]	Jaffar et al., 2008	48	F	No	Positive	Positive	Negative	-	-	Negative	-	3.3 cm	Lumpectomy
[30]	Jing et al., 2017	42	F	No	Positive	Positive	Positive	Positive	Negative	Positive	Negative	1.5 cm	Lumpectomy
[31]	Khatib et al., 2018	55	F	No	Positive	Positive	Positive	-	-	-	Negative	2 cm	Lumpectomy
[32]	Koufopoulos et al., 2022	37	F	No	Positive	Positive	Negative	-	Negative	Negative	Negative	3.2 cm	Lumpectomy
[33]	Laasri et al., 2022	69	M	-	Positive	-	Positive	Positive	-	Positive	Negative	25 cm	Lumpectomy
[34]	Omar et al., 2016	57	M	-	Positive	-	Positive	Positive	Negative	Positive	Negative	10 cm	Lumpectomy
[35]	Osment et al., 2022	69	M	No	Positive	-	-	-	Negative	Positive	-	2.9 cm	Lumpectomy
[36]	Qureshi et al., 2008	40	F	-	Positive	-	-	-	Negative	-	-	4 cm	Lumpectomy
[37]	Rochilis et al., 2017	50	M	Yes	Positive	-	-	-	-	Positive	-	0.8 cm	Lumpectomy
[38]	Scardina et al., 2021 (case 1)	80	M	No	-	-	-	-	-	Positive	-	3.6 cm	Lumpectomy
[38]	Scardina et al., 2021 (case 2)	59	F	No	Negative	-	Negative	-	Negative	Positive	Negative	2 cm	Lumpectomy
[39]	Uchoa et al., 2010	59	F	-	Positive	Positive	Negative	-	-	Positive	Negative	2.5 cm	Lumpectomy
[40]	Wang et al., 2003	72	M	No	Positive	Positive	Negative	-	Negative	Positive	Negative	3.3 cm	Lumpectomy
[41]	Wei et al., 2021	50	F	Yes	Negative	Positive	Positive	-	-	-	-	5 cm	Lumpectomy
[42]	Yang et al., 2020	42	F	No	Positive	-	Positive	Positive	Positive	Positive	-	2.5 cm	Lumpectomy
(-) indicates where a result was not specified in the report.													

**Table 2:** Comparison of patient presentation in 38 reported cases of myofibroblastoma of the breast.

Age		Sex	
Median	Range	Female	Male
59	17–82	24	14
Size		Associated pain	
Median	Range	Yes	No or unspecified
4 cm	0.2–25 cm	2	36
Treatment			
Lumpectomy	Unilateral mastectomy	Modified radical mastectomy	Vacuum-assisted
29	1	2	1

**Table 3:** Immunohistochemistry of 38 cases of myofibroblastoma of the breast.

	Positive	Negative	Unspecified
CD34	30	7	1
Vimentin	17	0	21
Smooth muscle actin	17	10	11
Estrogen receptor	16	1	21
Pancytokeratin	1	24	13
Desmin	25	4	9
S100	1	21	16

## Conclusions

The purpose of this report is to describe the variance in IHC among cases of MFB of the breast and to describe a case of MFB in a premenopausal woman. A detailed case review of 38 MFB case reports portrays the challenges in diagnosing MFB of the breast, as a result of the variety in symptom presentation, age, gender, location, and IHC staining. This denotes the importance of proper workup of this lesion, including proper IHC analysis to form a diagnosis, which can sometimes have pitfalls. As most of the cases that were reviewed reported surgical excision of the MFB masses, their recurrence, implications, complications, and malignant transformations have not been studied over the long term. Future research could be conducted on cases where a diagnosis of MFB was performed before the excision of the breast masses. Future research could also include a cost-to-benefit analysis comparing MFB excision via vacuum-assisted technique with excision versus lumpectomy in a large population.
